# Gut Microbiota Composition and *Clostridioides Difficile* Infection: The Potential Protective Role of *Faecalibacterium Prausnitzii*

**DOI:** 10.1080/29933935.2024.2390926

**Published:** 2024-09-05

**Authors:** Nadim Cassir, Jeanne Couturier, Johanne Delannoy, Auguste Wolfromm, Najiby Kassis Chikhani, Frédéric Barbut

**Affiliations:** a Université de Paris Cité, Institut national de la santé et de la recherche médicale (INSERM) UMR S-1139, Paris, France; b National Reference Laboratory for Clostridioides difficile, Assistance Publique-Hôpitaux de Paris (AP-HP), Saint-Antoine Hospital, Paris, France; c Équipe de Prévention du Risque infectieux, Hôpital Européen Georges Pompidou, Paris, France

**Keywords:** *Clostridioides difficile*, gut microbiota, *clostridioides difficile* infection, *clostridioides difficile* asymptomatic carriage, *Faecalibacterium prausnitzii*, *clostridium scindens*

## Abstract

The gut microbiota plays a pivotal role in Clostridioides *difficile* infection (CDI). We aimed to compare the composition of the gut microbiota of patients with CDI, patients asymptomatically colonized by Clostridioides *difficile* (CDC), patients with non-C. *difficile* diarrhea (NCD), and healthy controls (HCs) using 16S rRNA gene amplicon sequencing. We included 12 patients with CDI. Each CDI case was matched with four of CDC, including two with toxigenic CD strains, two patients with NCD, and two HC. Patients in each group were matched by age subgroup and antibiotic exposure. Bacterial richness was significantly higher in the HC group than in the CDC, NCD, and CDI groups. Beta-diversity analysis of the gut microbiota showed the Unifrac index to differ significantly between all groups. Relative to all other groups, the HC gut microbiota was significantly enriched by *Faecalibacterium prausnitzii and Sporobacter* sp. *Mediterranibacterium torques, Campylobacter hominis, Blautia* sp. *Mobiluncus curtisii*, and members of the Lachnospiraceae family. The most significantly depleted taxa in the gut microbiota of patients with CDI versus that of the HC group was *F. prausnitzii*. Our findings argue in favor of a potential protective role of *F. prausnitzii* against CDI, independently of age and antibiotic exposure.

## Introduction

*Clostridioides difficile* (CD) is the leading cause of antibiotic-associated colitis, responsible for clinical presentations ranging from mild diarrhea to life-threatening toxic megacolon. CD is considered to be the major agent of healthcare-associated diarrhea, leading to prolonged hospital stays, higher mortality rates, and substantial costs.^1^ In France, the incidence of *Clostridioides difficile* infection (CDI) in acute care has increased over the last 20 years, reaching 3.6 cases per 10,000 patient days in 2016.^2^

The asymptomatic CD carriage rate is estimated to be between 4% and 15% among healthy adults and up to 26% among healthcare-exposed patients.^3^ In a recent French point prevalence study involving 11 hospitals, the prevalence of toxigenic CD carriage among hospitalized patients was 3.5% for patients aged >3 years and 7.0% for those aged ≤3 years.^4^ Adult patients colonized with a toxigenic strain have a higher CDI risk than noncolonized patients.^5^

The interactions between CD and the gut microbiota significantly contribute to the development of CDI. Indeed, the most important risk factor for CDI is antibiotic use,^6,7^ which results in intestinal dysbiosis. Fecal microbiota transplantation (FMT) restores gut microbiota diversity and is associated with the clinical cure of recurrent CDI in >90% of cases, which is significantly higher than antibiotic therapies targeting CD.^8^ The commensal gut microbiota composition also plays a pivotal role in protection against CD acquisition, also called colonization resistance.^9^ Metabolic pathways with relevance to CD germination have been identified, one of the best characterized being the conversion from primary to secondary bile acids. Indeed, it has been shown that bacteria producing 7α-dehydroxylated secondary bile acids may protect against *C. difficile* vegetative growth and CDI.^10^ Previous studies using various techniques, from culture-based methods to high-throughput sequencing, have shown lower diversity and alterations of the gut microbiota composition in adult CDI patients than healthy controls or asymptomatic CD carriers.^7,11–22^ However, recent studies have shown that gut microbiota diversity and composition could, in turn, be influenced by the presence of CD strains in adults and children.^23,24^

We aimed to compare the composition of the gut microbiota of CDI patients, patients asymptomatically colonized by CD, patients with non-CD diarrhea, and healthy controls (HCs) (*i.e.*, without diarrhea) using sequencing of the V3-V4 region of the 16S rRNA gene. Patients in each group were matched by age subgroup and antibiotic exposure.

## Materials and methods

### Study design and setting

This is an ancillary study of the CODBAHRE project,^4,25^ a point-prevalence study to estimate multidrug-resistant organisms and CD carriage rates performed in 11 healthcare facilities of the Assistance Publique-Hôpitaux de Paris (AP-HP) in France.

This study was approved according to French regulations (AP-HP project CODBAHRE no. 180561; IDRCB no. 2019-A01226-51).

A non-opposition form was obtained from each patient included in the study (or parents/guardians for pediatric patients). A questionnaire including demographic data and information on any antibiotic treatment in the month prior to sampling was completed.

For all included patients, a screening sample was taken using the eSwab® system (COPAN, Brescia, Italy) by a nurse or the patients themselves. The sample was collected by rectal swabbing or soaking the swab in the stools. All samples were sent to a central microbiology laboratory for subsequent analysis. The epidemiological results of this study were presented elsewhere.^4^ Among the 2,389 patients included, 185 (7.7%) had a *C. difficile*-positive sample.

### Patient groups

We included 12 patients with a CDI, defined as patients with diarrhea (≥3 unformed stools in 24 h) whose stool samples were positive for CD and toxin genes but negative for other pathogens.^26^ Each case patient was matched with four asymptomatic CD carriers (CDC), including two with toxigenic CD strains, two with non-*C. difficile* diarrhea (NCD), and two HCs **(Figure S1)**. Patients with NCD were defined as patients with diarrhea (≥3 unformed stools in 24 h) whose stool samples were negative for CD and toxin genes but possibly positive for other pathogens. Patients in each group were matched by age subgroup (<3, 3–18, 18–65, and >65 years of age) and antibiotic exposure during a full course of treatment in the last 6 months. CDC patients had a positive stool sample with toxigenic or non-toxigenic CD strains but did not exhibit any diarrhea or other gastrointestinal disorders at the time of stool collection.^27^ All CDI episodes included in the study were primary, and no previous CDI was recorded in the controls (CDC, HC, and NCD).

#### Clostridioides difficile isolation

*difficile* isolation was performed as previously described.^28^

### 16S rRNA sequencing and analysis

The V3 to V4 hypervariable regions of the 16S rRNA gene were amplified by PCR as previously described.^4^ Paired-end reads were analyzed using the pipeline “Find Rapidly OTU with Galaxy Solution” (FROGS) version 4.1.^29^ Reads of 380–500 bp were retained for processing. The clustering and chimera removal tools followed the FROGS guidelines. Amplicon sequence variants (ASVs) corresponded to aggregated sequences at the taxonomic level with at least 99% similarity (identity and coverage). ASVs with an abundance <0.005% of all sequences were removed from the analysis.^30^ Assignment was performed using the Silva 138.1 pintail100 database and RDP classifier algorithm (http://rdp.cme.msu.edu/accessed 09/11/2023).

Taxonomic affiliation was verified using BLAST (NCBI standard nucleotide blast https://blast.ncbi.nlm.nih.gov/Blast.cgi, accessed 09/11/2023) by comparing the ASV nucleotide sequence with the RefSeq_rna database. Taxonomic affiliation was considered “exact” if the percentage of identity was 100%.

16S sequencing data were analyzed using the R environment version 4.3.2 (R Foundation) and phyloseq, microbiome, microbiomeMarker, and MicrobiotaProcess packages, in addition to custom scripts, as previously described.^31^ All sequence data in this study were uploaded to the NCBI Sequence Read Archive (SRA) database (Accession Number: PRJNA1062587).

### Biodiversity and Microbial Community Analysis

To adjust for the influence of uneven sampling depth, each sample was rarefied to a sampling depth of 27,246 reads, leading us to discard two samples. Species richness (observed ASVs, Chao-1) and α-diversity (Shannon index, inverse Simpson index) were analyzed using the phyloseq package (v1.46.0). Beta-diversity indexes assessing gut microbiota dissimilarities between patient groups were measured using the Jaccard, Bray-Curtis, Unifrac, and weighted UniFrac distance matrices and were used to build principal coordinate analysis (PCoA) plots. The Bray-Curtis dissimilarity distance reflects community composition by considering the abundance of taxa, while the UniFrac distance considers the phylogenetic relationships among members of bacterial communities. The Venn diagram to assess community overlaps was created by MicrobiotaProcess (v1.14.0) and VennDiagram (v1.7.3). Taxa associated with each group were determined by the linear discriminant analysis (LDA) effect size (LefSe) algorithm by microbiomeMarker (v1.7.0).^32^ The degree of enrichment was proportional to the LDA score. Only taxa with a LDA score >3 are displayed.

### Statistical analysis

All statistical analyses were performed in R (4.3.2). Results with a p-value <0.05 were considered significant. Differences between groups were determined using Chi-2 tests or Fisher’s exact test when appropriate. We used non-parametric ANOVA (Kruskal–Wallis test) to compare α-diversity indexes between patient groups, followed by pairwise group analysis using t tests. Permutational multivariate analysis of variance (PERMANOVA) was performed using the “adonis” function with 999 permutations and Unifrac distances to separately investigate the associations between microbiota composition confounding variables (sex, age, and antibiotic exposure). For the LEfSe analysis, the Kruskal–Wallis non-parametric factorial rank sum test was first performed, followed by pairwise group analysis using the unpaired Wilcoxon rank sum test. Linear discrimination analysis (LDA) was then used.

## Results

Among the 106 sequenced samples, we excluded two because the number of reads was outside the normal distribution. We then analyzed and compared the gut microbiota composition of 104 patients: 12 had CDI, 22 were control patients without diarrhea, 47 were asymptomatically colonized with *C. difficile*, and 23 had diarrhea without *C. difficile* (**Figure S1**).

Patients in each group were matched by age subgroup (<3, 3–18, 18–65, and >65 years of age) and antibiotic exposure (Table 1). Rarefaction curves of the datasets presented by the group are available in Supplementary Material (**Figure S2**). In total, 540 ASVs belonging to 146 genera in 60 families and eight phyla were identified.

**Table 1.** Patient characteristics.

	HC N = 22	CDC N = 47	CDI N = 12	NCD N = 23	P
Sex ratio (M/F)	0.69	1.61	0.71	0.92	
Age group N (%)					
0-3 yo	7 (32%)	16 (34%)	3 (25%)	7 (30%)	0.97
3-18 yo	1 (4.5%)	4 (8.5%)	2 (17%)	2 (9%)	0.68
18-65 yo	8 (36%)	15 (32%)	4 (33%)	8 (35%)	0.98
>65 yo	6 (27%)	12 (26%)	3 (25%)	6 (26%)	0.99
Previous antibiotics N (%)	18 (82%)	38 (81%)	9 (75%)	17 (74%)	0.85

HC: healthy controls, CDC: asymptomatic *C. difficile* carriers, CDI: *C. difficile* infection, NCD: patients with diarrhea without *C. difficile*, yo: years old.

*C. difficile* ASVs were only detected in 33/60 (55%) patients with a positive *C. difficile* culture. The concordance rate between the culture and sequence-based technique was 60.4% (**Table S1**).

**Figure 1.**
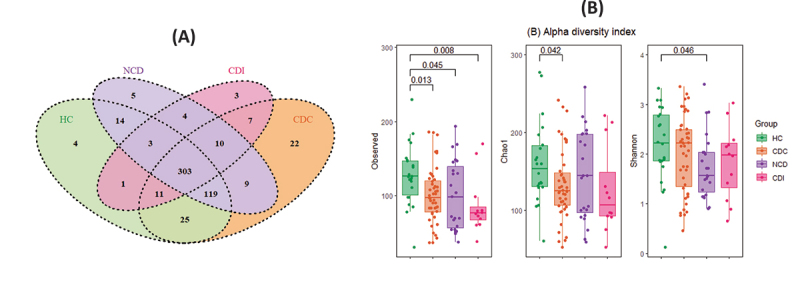
(A) overlapping amplicon sequence variants (ASVs) between groups displayed by a venn diagram. (B) Box plot of the alpha diversity (Shannon) and richness index (observed and Chao 1) for all groups of patients.

Overall, a difference was found between all groups in terms of richness (observed index: *p* = 0.028) but not in terms of diversity (Shannon index: *p* = 0.22). The observed index, reflecting the number of different taxa, was significantly higher in the HC group than the CDC (*p* = 0.013), NCD (*p* = 0.045), and CDI (*p* = 0.008) groups. In terms of the abundance-based estimation of species richness, the Chao1 index was significantly higher in the HC than CDC group (*p* = 0.042). In terms of α-diversity, the Shannon index was significantly higher in the HC than NCD group (*p* = 0.046), reflecting both greater richness and evenness within the bacterial community (Figure 1B). We found no significant differences in terms of richness or α-diversity between the CDI and CDC groups, although a trend toward lower richness and diversity indexes was observed in the CDI group.

ASVs were designated by a genetic distance threshold of 0.01. The comparison of microbial communities between groups was made using pooled samples. We used non-parametric ANOVA (Kruskal–Wallis test) to compare α-diversity indexes between patient groups, followed by pairwise group analysis using t tests.

HC: healthy controls, CDC: asymptomatic C. *difficile* carriers, CDI: C. *difficile* infection, NCD: patients with diarrhea without C. *difficile*, ns: non-significant.

The number of ASVs in the CDI, CDC, NCD, and HC groups was 342, 506, 467, and 480, respectively. More than half of the total 540 ASVs were shared by all groups (Figure 1A).

**Figure 2.**
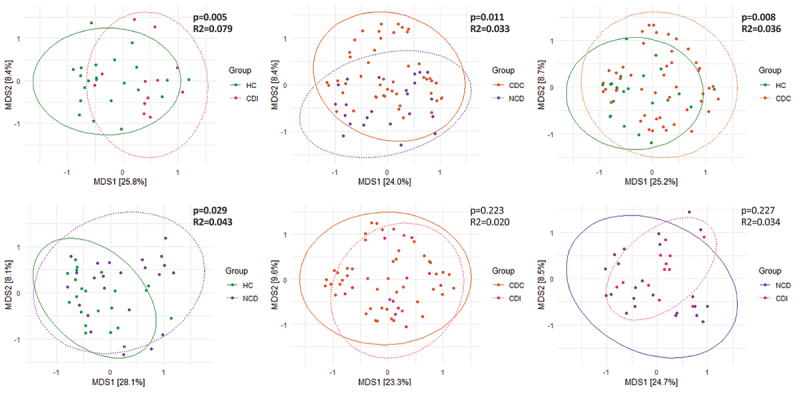
Beta-diversity: principal coordinate analysis (PCoA) based on unweighted unifrac distances of 16S rRNA gene sequences (V3-V4 region). Each axis represents inter-sample variation.

Beta-diversity analysis of the gut microbiota showed the Unifrac index to differ significantly (*p* = 0.02) between all groups, but not according to the Unifrac method, reflecting differences in the presence/absence of phylogenetically related bacterial taxa rather than in their relative abundance. The beta-diversity based on unweighted Unifrac distances of 16S rRNA gene sequences (V3-V4 region) was significantly different between the CDI and HC groups (*p* = 0.005) but not between CDI and CDC groups, nor between CDI and NCD groups. These results are represented by PCoA in Figure 2. The most important clinical factor associated with microbiota composition was the age group (PERMANOVA, *p* = 0.002, R^2^ = 0.075), followed by the patient group (PERMANOVA, *p* = 0.002, R^2^ = 0.060).

HC: healthy controls, CDC: asymptomatic *C. difficile* carriers, CDI: *C. difficile* infection, NCD: patients with diarrhea without *C. difficile*.

**Figure 3.**
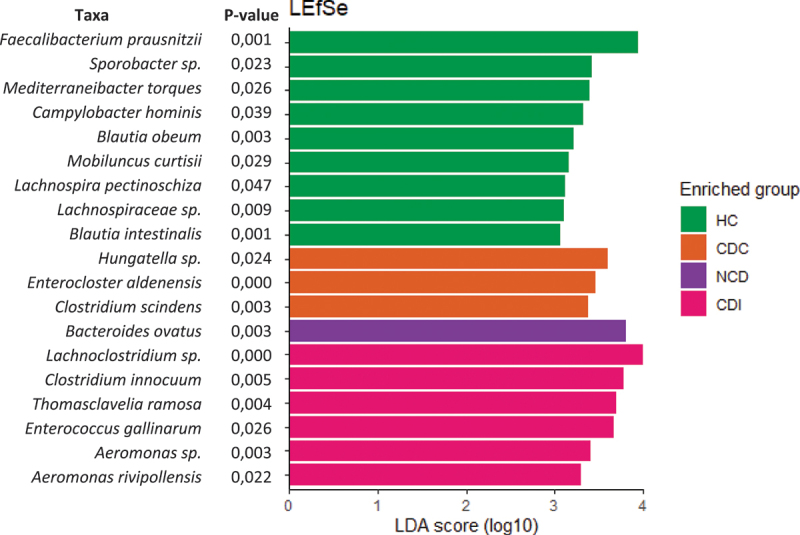
Linear discriminant analysis effect size (LEfSe) and linear discriminant analysis (LDA) based on amplicon sequence variants in all groups of patients. By analyzing the differential abundances, each group was compared to all other groups. Only taxa that met a significant difference (*p* < 0.05) and the LDA significance threshold > 3 are shown. The degree of enrichment is proportional to the LDA score.

There were large differences between the phylogenetic profiles of patients and groups **(Figure S3)**. We used LEfSe software to identify putative key microbial biomarkers by analyzing the differential abundances (Figure 3). Relative to all other groups, the gut microbiota of CDI patients was significantly enriched for *Lachnoclostridium* sp., *Clostridium innocuum, Thomasclavelia ramosa*, and *Aeromonas* species. Relative to all other groups, the gut microbiota of CDC patients was significantly enriched for *Hungatella* sp., *Enterocloster aldenensis*, and *Clostridium scindens*. Relative to all other groups, the gut microbiota of NCD patients was significantly enriched for *Bacteroides ovatus*. Relative to all other groups, the gut microbiota of HCs was significantly enriched for *Faecalibacterium prausnitzii, Sporobacter* sp., *Mediterranibacterium torques, Campylobacter hominis, Blautia* sp., *Mobiluncus curtisii, Anerotignum* sp., and members of the Lachnospiraceae family. Relative to the CDC group, the gut microbiota of CDI patients was significantly depleted of *Flavonifractor plautii, Enterocloster aldenensis, Oscillobacter* sp., and *Bacteroides* sp. (**Figure S4**). Relative to the HC group, the most significantly depleted taxa in the gut microbiota of CDI patients was *F. prausnitzii* (**Figure S5**).

HC: healthy controls, CDC: Asymptomatic *C. difficile* carriers, CDI: *Clostridium difficile* infection.

## Discussion

We compared the gut microbiota composition of CDI patients, patients asymptomatically colonized by CD, patients with non-*C. difficile* diarrhea, and healthy controls. Although the Shannon index showed no differences, the taxa richness was significantly higher in the HC than in the CDC group (Figure 1). We found no significant differences in terms of richness or α-diversity between the CDI and CDC groups, although there was a trend toward lower richness and diversity indexes in the CDI group. These results are in accordance with available data from 16S rRNA gene NGS analysis suggesting that *C. difficile* colonization or infection is associated with lower richness and diversity of the gut microbiota in adults and children than in healthy controls.^33^ Crobach MJT et al. found in a similar analysis that bacterial diversity was significantly decreased in CDC and CDI patients, while the overall microbiota composition was significantly different between control, CDC, and CDI patients.^21^ In accordance with previous studies, we found no differences in terms of diversity between the CDI and NCD groups.^13,14,20^ However, other previous studies reported a lower ⍺-diversity for patients with CDI than those developing nosocomial diarrhea without CD.^15,16^ Notably, in our study, patients in the HC, CDC, and NCD groups were matched with the CDI group by antibiotic exposure. Differences in the global composition of the gut microbiota were therefore less highly influenced by this confounding factor that leads to gut dysbiosis. However, we found a high inter-individual heterogeneity between and within groups (**Figure S3**) which could be partly explained by patients’ state of health and exposure to different classes of antibiotics (**Table S2**).

There is evidence that a certain level of richness and diversity of the gut microbiota is necessary to protect against CDI. However, *C. difficile* resistance appears to be due to more complex gut microbiota modifications. In particular, microbiota – produced butyric acid and other short-chain fatty acids play an important function in resisting pathogenic bacteria.^12^ Increased susceptibility to *C. difficile* carriage may be driven by the depletion of butyrate-producing bacteria. In accordance with previous reports,^12,34^ we found that *F. prausnitzii* was the most significantly depleted taxa in the gut microbiota of patients with CDI versus healthy controls. Notably, a recent study showed that successful FMT increases the relative abundance of *F. prausnitzii* in patients with recurrent CDI.^35^ Similarly, it has been shown that samples from patients with CDI had a significantly lower relative abundance of *Faecalibacterium* sp. than healthy controls.^12,13,16,19,20^ This suggests a protective role of *F. prausnitzii* against CDI. We also found significant enrichment of *Clostridium scindens* in samples from patients colonized with CD. Notably, secondary bile acids produced by *C. scindens* are known to inhibit *C. difficile* germination and growth.^10,36^ Recently, it has been shown that the *C. scindens* secretome can suppress CD toxin expression in a bile acid-independent manner.^37^ However, we did not find such enrichment between the CDI and CDC groups. Further studies on a larger scale are needed to confirm these results.

This study had several limitations. First, the number of patients included in each group was low, precluding the detection of small differences between their gut microbiota composition. In addition, we included three patients aged <3 years with a CDI diagnosis. Despite the high carrier state of young children, there are few cases of clinically documented CDI, especially in those with chronic medical conditions.^38^ Although patients were matched by age group, the most important clinical factor associated with microbiota composition was the age group, increasing the differences within each group. Other biases could have altered the results of the comparative gut microbiota analysis in terms of diversity and composition. For example, differences in feeding or pump inhibitor use could also be confounding factors.^39,40^ Second, we did not test for free toxins as recommended,^41^ because swabs were tested after the search for MDROs, with sub-optimal storage conditions and the associated risk of degradation of free toxins. Third, the concordance rate between culture- and sequence-based techniques for CD detection was only 60.4%, which is lower than that reported by previous studies.^21,31^ It has been shown that NGS-based methods can miss the detection of some bacterial species in the adult gut microbiota at concentrations <10^5^ CFU/mL.^42^ However, the detection limits of NGS-based methods vary between studies, notably because of the use of different primers targeting different regions of the 16S rRNA gene. Furthermore, the detection of the 16S rRNA gene does not indicate the viability of CD in the sample. In addition, we previously found that the CD detection threshold for the culture-based method was 3.3 log_10_ CFU/g stool.^43^ Altogether, we cannot exclude the possibility of misclassification in each group. Finally, our analysis was based on ≤500-bp 16S partial rRNA gene sequences, which may have misclassified certain bacteria at the species or strain level. All taxonomic affiliations were verified using BLAST (NCBI standard nucleotide blast https://blast.ncbi.nlm.nih.gov/Blast.cgi., accessed 09/11/2023) and were retained at each taxonomic level only when the percentage of identity was 100%. Nonetheless, as for most studies using NGS of the 16S rRNA, our results are restricted by low precision at the taxonomic level.

## Conclusions

We found several bacterial taxa that could be used as biomarkers to differentiate the gut microbiota composition between patients with CDI, patients asymptomatically colonized by CD, patients with non-CD diarrhea, and healthy controls, independently of age and antibiotic exposure. Notably, our findings argue in favor of a potential protective role of *F. prausnitzii* against CDI.

## Supplementary Material

Supplemental Material

## Data Availability

The data that support the findings of this study are openly available in the NCBI database, with the reference number [**PRJNA1062587**].
