# Supplementary material for: Gut Microbiota Composition and Clostridioides Difficile Infection: The Potential Protective Role of Faecalibacterium Prausnitzii
Source: Gut Microbes Rep. 2024 Sep 5;1(1):2390926. doi: 10.1080/29933935.2024.2390926 (PMC12940150; doi:10.1080/29933935.2024.2390926)
Supplement: Supplemental Material [file KGMR_A_2390926_SM3939.zip › FigS1.pdf]

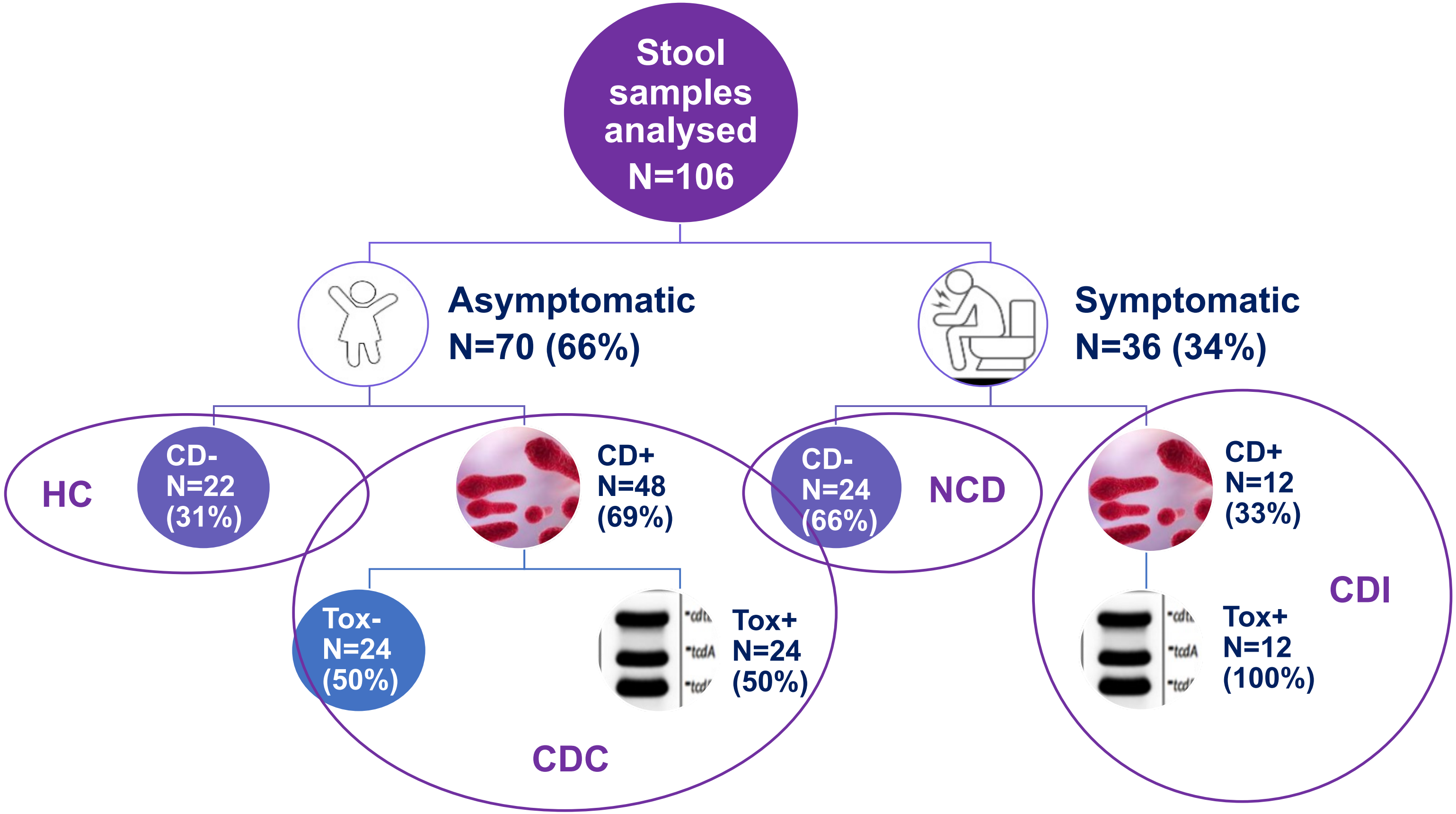
Stool samples analysed
N=106
Asymptomatic
N=70 (66%)
Symptomatic
N=36 (34%)
HC
CD-
N=22
(31%)
CD+
N=48
(69%)
CD-
N=24
(66%)
NCD
CD+
N=12
(33%)
Tox-
N=24
(50%)
Tox+
N=24
(50%)
CDC
CDI
Tox+
N=12
(100%)
