# Supplementary figures and images for: Gut Microbiota Composition and Clostridioides Difficile Infection: The Potential Protective Role of Faecalibacterium Prausnitzii
Source: Gut Microbes Rep. 2024 Sep 5;1(1):2390926. doi: 10.1080/29933935.2024.2390926 (PMC12940150; doi:10.1080/29933935.2024.2390926)

Species Richness

HC

CDC

NCD

CDI

Group

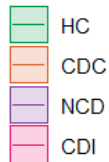

Sample Size

200  
150  
100  
50  
0

0

10000

20000

0

10000

20000

Supplement: Supplemental Material [file KGMR_A_2390926_SM3939.zip › FigS2.pdf]

Relative abundance

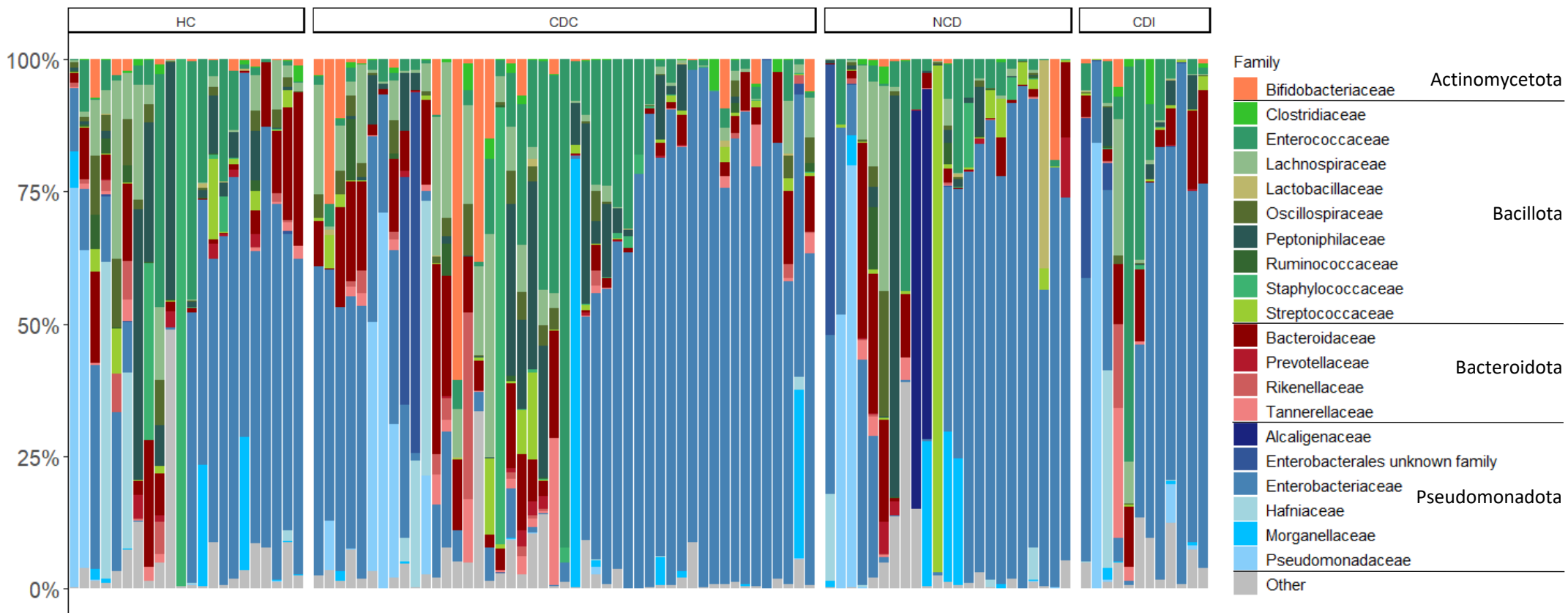

Supplement: Supplemental Material [file KGMR_A_2390926_SM3939.zip › FigS3.pdf]

## LEfSe

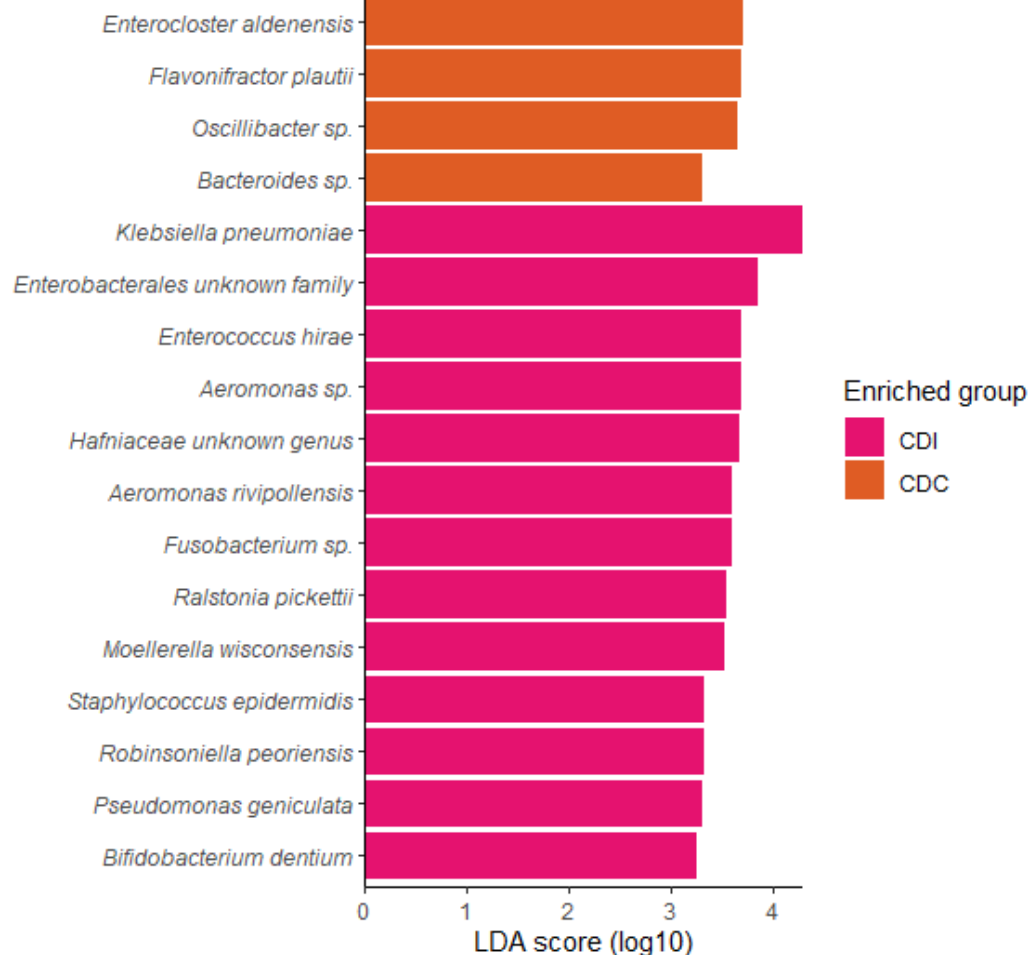

Supplement: Supplemental Material [file KGMR_A_2390926_SM3939.zip › FigS4.pdf]

## LEfSe

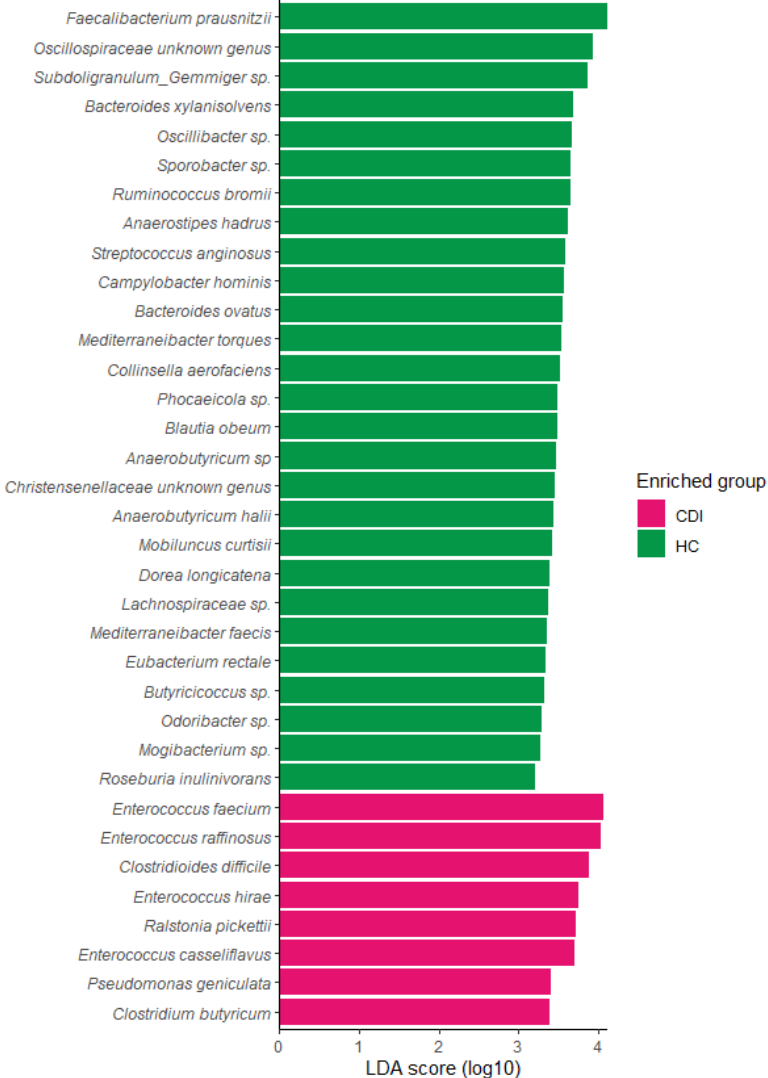

Supplement: Supplemental Material [file KGMR_A_2390926_SM3939.zip › FigS5.pdf]
